# Make Data Sharing Routine to Prepare for Public Health Emergencies

**DOI:** 10.1371/journal.pmed.1002109

**Published:** 2016-08-16

**Authors:** Jean-Paul Chretien, Caitlin M. Rivers, Michael A. Johansson

**Affiliations:** 1 Integrated Biosurveillance Section, Armed Forces Health Surveillance Branch, Defense Health Agency, Silver Spring, Maryland, United States of America; 2 Epidemiology and Disease Surveillance, US Army Institute of Public Health, Aberdeen Proving Ground, Maryland, United States of America; 3 Dengue Branch, Division of Vector-Borne Diseases, Centers for Disease Control and Prevention, San Juan, Puerto Rico, United States of America

## Abstract

Jean-Paul Chretien and colleagues argue that recent Ebola and Zika virus outbreaks highlight the importance of data sharing in scientific research.

**Summary Points**

- The recent outbreaks caused by Ebola and Zika viruses highlighted the importance of medical and public health research in accelerating outbreak control and prompted calls for researchers to share data rapidly and widely during public health emergencies.
- Effective preparation for emergencies requires the routine practice of data sharing in scientific research.
- Key impediments to data sharing, such as long-standing academic norms and human and technical resource limitations, cannot immediately be surmounted when an emergency occurs.
- Ongoing research that does not directly relate to an emergency now may be critical for the next unpredictable outbreak.
- As part of emergency preparedness, the scientific community should support ongoing initiatives that address major obstacles to data sharing and should embrace open science practices in both emergency and nonemergency research.

In February 2016, Wellcome Trust organized a pledge among leading scientific organizations and health agencies encouraging researchers to release data relevant to the Zika outbreak as rapidly and widely as possible [1]. This initiative echoed a September 2015 World Health Organization (WHO) consultation that assessed data sharing during the recent West Africa Ebola outbreak and called on researchers to make data publicly available during public health emergencies [2]. These statements were necessary because the traditional way of communicating research results—publication in peer-reviewed journals, often months or years after data collection—is too slow during an emergency.

The acute health threat of outbreaks provides a strong argument for more complete, quick, and broad sharing of research data during emergencies. But the Ebola and Zika outbreaks suggest that data sharing cannot be limited to emergencies without compromising emergency preparedness. To prepare for future outbreaks, the scientific community should expand data sharing for all health research.

## Open Science, Ebola, and Zika

Recent calls for data sharing during public health emergencies can be viewed as part of a broader movement towards open science (Box 1).

**Box 1. Open Science**

Various definitions of **open science** converge on the concept of unlimited access to all aspects of research, to allow anyone to follow, use, and participate in science. **Open science** comprises a growing list of other “opens,” such as **open access** (scholarly literature not only is freely available online but may be reproduced, distributed, and otherwise reused by others, typically according to the terms of a public copyright license that accompanies the article); **open data** (data, including data underlying publications, are freely available online and may be used and shared); and **open source** (software is freely available and may be modified and distributed) [3–5]. Sometimes, scientific products meet criteria for open access, open data, or open source after some time has elapsed since their production (e.g., “delayed open access” journals make content available only to subscribers initially and then make it open access later, typically after 1–2 years). An expansive version of open science is **open notebook science**, in which the entire primary record of research, including the researcher’s personal or laboratory notebook, is freely available online as it is recorded [6]. **Open government** aims to improve citizens’ access to government data and proceedings [7] and advances open science, especially for government-funded research.

In the health sciences, an important milestone for openness was achieved 20 years ago, as genetic sequencing began to generate massive amounts of data and scientists agreed to deposit sequences in public databases almost as they were produced. Encouraged by the discoveries this facilitated, life science leaders convened summits that extended the call for openness to other types of datasets [8]. Major public health research funders agreed to increase the availability of research data and to promote the use of those data to accelerate advances in public health [9]. Today, the movement towards open science is evident across the health sciences landscape (Box 2), including recent emergencies.

**Box 2. Examples of Increasing Openness in Health Sciences**

Funding agencies: Major research sponsors have implemented policies that encourage or require data sharing. In 2003, the United States National Institutes of Health (NIH) began requiring a data-sharing plan for grant applications with annual costs over US$500,000; a 2013 national survey found that 65% of life science researchers thought the NIH policies had been influential in increasing data sharing [10]. The US Centers for Disease Control and Prevention also adopted a data-sharing policy in 2003 [11], more recently requiring grantees to include a data release plan [12]. The Bill & Melinda Gates Foundation, beginning in 2017, will require peer-reviewed publications and underlying data to be open immediately on publication [13]. Open government initiatives [14] are increasing public access to government-held data, including data collected in scientific research.

Scientific journals: The proportion of articles indexed in PubMed that were freely available online within about a year of publication nearly doubled from 2006 to 2010, from 26% to 50% [15]. A search of the Scopus database in April 2014 estimated that 71% of biomedical research papers published during 2011–2013 were freely available online (though only about a quarter of these were made available immediately on publication by the publisher or author) [16]. At the same time, several prominent journals now encourage data sharing and require a statement about data accessibility [17]; PLOS, beginning in 2014, required authors to make all underlying data publicly available on publication for its family of journals. In 2016, the International Committee of Medical Journal Editors (ICMJE) proposed the requirement that authors submitting clinical trial reports to ICMJE member journals make the deidentified individual patient data underlying the study available within 6 months of publication [18].

Scientists: Some individual researchers and institutions have adopted nearly comprehensive openness. For example, to accelerate discovery in neuroscience, the Montreal Neurological Institute and Hospital of McGill University is beginning an unprecedented 5-year experiment in openness during which it will make all data and results freely available and will not seek patents [19].

During the Ebola outbreak, researchers unaffiliated with official response efforts transformed surveillance reports into machine-readable formats and shared them in public repositories [20], and some teams assisting the response rapidly deposited Ebola virus genetic sequences into public databases [21]. These efforts allowed many scientists to contribute analytical insights—80% of peer-reviewed epidemiological modeling studies published during the outbreak used only open data [22]. Many researchers also shared computer code of their models online.

Pharmaceutical company leaders acknowledged that “depending on the circumstances for the emergency, preliminary data could be made available with clear descriptions of the verifications that are ongoing and the remaining risks to data integrity” [23]. WHO officials noted that research teams generated and exchanged critical data for novel vaccines faster than ever [24].

As the Zika epidemic highlighted major deficiencies in knowledge of the virus and disease, leading scientific journals agreed to make all Zika-related content free to access and not to penalize submissions for prepublication release of data or results [1]. Scientists organized a call for papers describing and releasing datasets related to Zika, to be considered for online publication in a peer-reviewed journal [25]. As during Ebola, scientists established a public repository for sharing Zika data [26]. One leading virology laboratory, inspired by rapid sharing of genomic data during the Ebola response, is releasing data from its animal model experiments with Zika virus online in real time [27].

## Data-Sharing Challenges

Despite these successes, the Ebola and Zika responses also highlight openness challenges for effective data sharing. Three major impediments limit data sharing and provide compelling reasons why emergency preparedness requires data sharing before emergencies occur.

First, there are no established standards for data users to credit data providers. In one example, researchers in Brazil who deposited Zika virus genome sequences in a public database felt they were not credited appropriately when another group used those sequences for a paper published 2 weeks later [28].

The scientific community has not yet established standards that could have prevented the disagreement. In one survey of clinical and basic science researchers, 50% of those who shared data were not credited in any way in the resulting publication or were recognized only in the acknowledgments section [29]. Opinions diverge over whether data providers should review results before publication, collaborate on the analysis, approve the analysis plan in advance, or limit conditions of data reuse [30]. Community-wide standards are needed so that the risk of uncredited secondary analysis will not dissuade scientists from sharing.

Second, scientists may doubt that sharing data will advance their scholarly stature as much as publishing primary research. During the Ebola response, some researchers waited weeks or months before releasing Ebola virus genomic data [21]. Their motivations are unknown, but fear of granting a competitive advantage to other scientists is a deterrent to sharing in the usual course of scientific research [31] and likely explains some data-sharing failures during the outbreak [32].

In a national US survey, 28% of life scientists reported intentionally delaying publication by more than 6 months to protect scientific primacy or for other nontechnical reasons. Some of them may have drawn lessons from experience: 25% of those who had shared data, information, or materials reported they had been “scooped” by another scientist [33]. A *PLOS Medicine* editorial succinctly summarized the challenge, which applies in emergency and nonemergency settings: “as long as authorship of individual published reports is perceived to confer greater reward than generating and sharing the data that underlie them, a disincentive to share data will persist” [34].

Third, scientists may not be able to share data effectively because of inadequate technology, standards, or human capacity. One of the reasons researchers could share genetic sequences effectively during the Ebola and Zika outbreaks, besides longstanding openness norms in the community, was their familiarity with public databases designed for such data (e.g., GenBank). Widely accepted central databases do not exist for other types of research data. Clinical trial data, for example, mostly reside in independent databases and are collected under various standards [35]. Some platforms are little more than “data dumpsters” without the metadata, data dictionaries, or documentation required for responsible analysis [36]. Any data-sharing arrangement faces the challenge of protecting patient privacy while preserving the usefulness of the data shared, a topic of active methodological research.

Obstacles are even more significant in lower-resource settings [37]. A review of the Ebola response found that affected countries lacked integrated standards for data collection and that “data were not aggregated, analyzed, or shared in a timely manner and in some cases not at all” [38]. In Sierra Leone, for example, inadequate standards allowed a date to refer ambiguously to when data was collected, submitted, or edited [39]. Sharing data in a useful way requires staff time, technical infrastructure, and human capacities that may not be available in low-resource settings. These essential elements of effective data sharing cannot be expected to materialize during a crisis.

## Preparing for the Next Surprise

Open data deserves recognition and support as a key component of emergency preparedness. Initiatives to facilitate discovery of datasets and track their use [40–42]; provide measures of academic contribution, including data sharing that enables secondary analysis [43]; establish common platforms for sharing and integrating research data [44]; and improve data-sharing capacity in resource-limited areas [45] are critical to improving preparedness and response.

Research sponsors, scholarly journals, and collaborative research networks can leverage these new opportunities with enhanced data-sharing requirements for both nonemergency and emergency settings. A proposal to amend the International Health Regulations with clear codes of practice for data sharing warrants serious consideration [46]. Any new requirements should allow scientists to conduct and communicate the results of secondary analyses, broadening the scope of inquiry and catalyzing discovery. Publication embargo periods, such as one under consideration for genetic sequences of pandemic-potential influenza viruses [47], may lower barriers to data sharing but may also slow the timely use of data for public health.

Integrating open science approaches into routine research should make data sharing more effective during emergencies, but this evolution is more than just practice for emergencies. The cause and context of the next outbreak are unknowable; research that seems routine now may be critical tomorrow. Establishing openness as the standard will help build the scientific foundation needed to contain the next outbreak.

Recent epidemics were surprises—Zika and chikungunya sweeping through the Americas; an Ebola pandemic with more than 10,000 deaths; the emergence of severe acute respiratory syndrome and Middle East respiratory syndrome, and an influenza pandemic (influenza A[H1N1]pdm09) originating in Mexico—and we can be sure there are more surprises to come. Opening all research provides the best chance to accelerate discovery and development that will help during the next surprise.

## Abbreviations

ICMJE: International Committee of Medical Journal Editors
NIH: National Institutes of Health
WHO: World Health Organization

## References

[1] Wellcome Trust. Sharing data during Zika and other global health emergencies. 10 Feb 2016. https://wellcome.ac.uk/news/sharing-data-during-zika-and-other-global-health-emergencies

[2] World Health Organization. Developing global norms for sharing data and results during public health emergencies. http://www.who.int/medicines/ebola-treatment/data-sharing_phe/en/ 10.1371/journal.pmed.1001935 PMC4701443 26731342

[3] Amsen E. What is open science? Discussions–F1000 Research. http://blog.f1000research.com/2014/11/11/what-is-open-science/

[4] Hanwell M. What is open science? Opensource.com. https://opensource.com/resources/open-science

[5] Pomerantz J, Peek R. Fifty shades of open. First Monday. 2016;21. http://firstmonday.org/ojs/index.php/fm/article/view/6360

[6] Open notebook science. Wikipedia, the free encyclopedia. https://en.wikipedia.org/w/index.php?title=Open_notebook_science&oldid=719360582

[7] Open government. Wikipedia, the free encyclopedia. https://en.wikipedia.org/w/index.php?title=Open_government&oldid=681917606

[8] Toronto International Data Release Workshop Authors, Birney E, Hudson TJ, Green ED, Gunter C, Eddy S, et al Prepublication data sharing. Nature. 2009;461: 168–170. 10.1038/461168a 19741685 PMC3073843

[9] Walport M, Brest P. Sharing research data to improve public health. Lancet 2011;377: 537–539. 10.1016/S0140-6736(10)62234-9 21216456

[10] Pham-Kanter G, Zinner DE, Campbell EG. Codifying collegiality: recent developments in data sharing policy in the life sciences. PLoS ONE. 2014;9: e108451 10.1371/journal.pone.0108451 25259842 PMC4178158

[11] Centers for Disease Control and Prevention/Agency for Toxic Substances and Disease Registry. CDC/ATSDR policy on releasing and sharing data. 16 April 2003 (updated 7 September 2005). http://www.cdc.gov/maso/Policy/ReleasingData.pdf

[12] Centers for Disease Control and Prevention. Additional requirements for funding opportunity announcements. AR-25: Release and sharing of data. http://www.cdc.gov/grants/additionalrequirements/index.html#ui-id-49

[13] Bill & Melinda Gates Foundation. Open Access Policy. http://www.gatesfoundation.org/How-We-Work/General-Information/Open-Access-Policy

[14] Open Government Partnership. http://www.opengovpartnership.org/

[15] Kurata K, Morioka T, Yokoi K, Matsubayashi M. Remarkable growth of open access in the biomedical field: analysis of PubMed articles from 2006 to 2010. PLoS ONE. 2013;8: e60925 10.1371/journal.pone.0060925 23658683 PMC3641021

[16] Archambault E, Amyot D, Deschamps P, Nicol A, Provencher D, Rebout L, Roberge G. Proportion of open access papers published in peer-reviewed journals at the European and world levels—1996–2013. European Commission. 2014. http://science-metrix.com/en/publications/reports/proportion-of-open-access-papers-published-in-peer-reviewed-journals-at-the

[17] Barbui C. Sharing all types of clinical data and harmonizing journal standards. BMC Med. 2016;14: 63 10.1186/s12916-016-0612-8 27038634 PMC4818917

[18] Taichman DB, Backus J, Baethge C, Bauchner H, de Leeuw PW, Drazen JM, et al Sharing Clinical Trial Data: A Proposal From the International Committee of Medical Journal Editors. Ann Intern Med. 2016;164: 505–506. 10.7326/M15-2928 26792258

[19] Owens B. Data Sharing. Montreal institute going “open” to accelerate science. Science. 2016;351: 329 10.1126/science.351.6271.329 26797995

[20] cmrivers/ebola. GitHub. https://github.com/cmrivers/ebola

[21] Yozwiak NL, Schaffner SF, Sabeti PC. Data sharing: Make outbreak research open access. Nature. 2015;518: 477–479. 10.1038/518477a 25719649

[22] Chretien J-P, Riley S, George DB. Mathematical modeling of the West Africa Ebola epidemic. eLife. 2015;4 10.7554/eLife.09186 PMC4769159 26646185

[23] Vallance P, Freeman A, Stewart M. Data Sharing as Part of the Normal Scientific Process: A View from the Pharmaceutical Industry. PLoS Med. 2016;13: e1001936 10.1371/journal.pmed.1001936 26731493 PMC4701406

[24] Modjarrad K, Moorthy VS, Millett P, Gsell P-S, Roth C, Kieny M-P. Developing Global Norms for Sharing Data and Results during Public Health Emergencies. PLoS Med. 2016;13: e1001935 10.1371/journal.pmed.1001935 26731342 PMC4701443

[25] Messina J, Kraemer M, Hay S. Call for submissions: Zika virus related datasets Scientific Data 20 1 2016 http://blogs.nature.com/scientificdata/2016/01/20/call-for-submissions-zika-virus-related-datasets/

[26] cdcepi/zika. GitHub. https://github.com/cdcepi/zika

[27] Butler D. Zika researchers release real-time data on viral infection study in monkeys. Nature. 2016; 10.1038/nature.2016.19438

[28] Callaway E. Zika-microcephaly paper sparks data-sharing confusion. Nature. 2016; 10.1038/nature.2016.19367

[29] Federer LM, Lu Y-L, Joubert DJ, Welsh J, Brandys B. Biomedical Data Sharing and Reuse: Attitudes and Practices of Clinical and Scientific Research Staff. PLoS ONE. 2015;10: e0129506 10.1371/journal.pone.0129506 26107811 PMC4481309

[30] Tenopir C, Dalton ED, Allard S, Frame M, Pjesivac I, Birch B, et al Changes in Data Sharing and Data Reuse Practices and Perceptions among Scientists Worldwide. PLoS ONE. 2015;10: e0134826 10.1371/journal.pone.0134826 26308551 PMC4550246

[31] Smith R, Roberts I. Time for sharing data to become routine: the seven excuses for not doing so are all invalid. F1000Research. 2016;5: 781 10.12688/f1000research.8422.1 27347380 PMC4909097

[32] Whitty CJM, Mundel T, Farrar J, Heymann DL, Davies SC, Walport MJ. Providing incentives to share data early in health emergencies: the role of journal editors. Lancet. 2015;386: 1797–1798. 10.1016/S0140-6736(15)00758-8 26843294 PMC7136984

[33] Zinner DE, Pham-Kanter G, Campbell EG. The Changing Nature of Scientific Sharing and Withholding in Academic Life Sciences Research: Trends From National Surveys in 2000 and 2013. Acad Med J Assoc Am Med Coll. 2016;91: 433–440. 10.1097/ACM.0000000000001028 PMC4767624 26675188

[34] PLOS Medicine Editors. Can Data Sharing Become the Path of Least Resistance? PLoS Med. 2016;13: e1001949 10.1371/journal.pmed.1001949 26812392 PMC4727892

[35] Berlin JA, Morris S, Rockhold F, Askie L, Ghersi D, Waldstreicher J. Bumps and bridges on the road to responsible sharing of clinical trial data. Clin Trials. 2014;11: 7–12. 10.1177/1740774513514497 24408901

[36] Merson L, Gaye O, Guerin PJ. Avoiding Data Dumpsters—Toward Equitable and Useful Data Sharing. N Engl J Med. 2016; 10.1056/NEJMp1605148 27168351

[37] Bull S, Cheah PY, Denny S, Jao I, Marsh V, Merson L, et al Best Practices for Ethical Sharing of Individual-Level Health Research Data From Low- and Middle-Income Settings. J Empir Res Hum Res Ethics. 2015;10: 302–313. 10.1177/1556264615594606 26297751 PMC4547207

[38] World Health Organization. Report of the Ebola Interim Assessment Panel—May 2015. http://www.who.int/csr/resources/publications/ebola/ebola-interim-assessment/en/

[39] GovLab. Open Data’s Impact. http://odimpact.org/case-battling-ebola-in-sierra-leone.html

[40] Force 11 Data Citation Implementation Group. https://www.force11.org/group/data-citation-implementation-group

[41] bioCADDIE | biomedical and healthCAre Data Discovery and Indexing Ecosystem. https://biocaddie.org/

[42] Research Data Alliance. The DLI Service: an open one-for-all data-literature interlinking service. https://rd-alliance.org/dli-service-open-one-all-data-literature-interlinking-service.html

[43] Dinsmore A, Allen L, Dolby K. Alternative perspectives on impact: the potential of ALMs and altmetrics to inform funders about research impact. PLoS Biol. 2014;12: e1002003 10.1371/journal.pbio.1002003 25423184 PMC4244028

[44] Bierer BE, Li R, Barnes M, Sim I. A Global, Neutral Platform for Sharing Trial Data. N Engl J Med. 2016; 10.1056/NEJMp1605348 27168194

[45] Carr D, Littler K. Sharing Research Data to Improve Public Health. J Empir Res Hum Res Ethics. 2015;10: 314–316. 10.1177/1556264615593485 26297752 PMC4547198

[46] McNabb SJN, Shaikh AT, Nuzzo JB, Zumla AI, Heymann DL. Triumphs, trials, and tribulations of the global response to MERS coronavirus. Lancet Respir Med. 2014;2: 436–437. 10.1016/S2213-2600(14)70102-X 24794576 PMC7129523

[47] World Health Organization. Handling of Influenza Genetic Sequence Data under the PIP Framework. http://www.who.int/influenza/pip/advisory_group/gsd/en

